# Editorial: Heart Rate Variability, Health and Well-Being: A Systems Perspective

**DOI:** 10.3389/fpubh.2019.00323

**Published:** 2019-11-05

**Authors:** Robert L. Drury, Stephen Porges, Julian Thayer, J. P. Ginsberg

**Affiliations:** ^1^ReThink Health, Cambridge, MA, United States; ^2^University of Wisconsin, Institute for Discovery, Madison, WI, United States; ^3^Kinsey Institute, Indiana University Bloomington, Bloomington, IN, United States; ^4^University of California, Irvine, Irvine, CA, United States; ^5^Wm. Jennings Bryan Dorn VA Medical Center, Columbia, SC, United States

**Keywords:** heart rate variability (HRV), integrative health, autonomic nerve activity, cardiac control, coherence

The Guest Editors of this Frontiers Research Topic are excited at the interest that our collection of articles has generated so far and the evident contribution to the field that this publication has had and will have. The topic co-editors have all been involved in its successful production, each with respect to his particular strength. We gratefully acknowledge the patient and thoughtful effort 70 contributors who are responsible for doing the hard work necessary to make this achievement possible.

The impact from 130,000 views of our Research Topic as of May 2019 is a testament to the power that the Frontiers platform offers scientists, researchers, clinicians, and authors to get their work out in front of readers. This special issue is put into large-scale perspective by the opinion piece by Grippo who gives us an overview of the commonality of comparative mammalian behaviors that HRV can be used to characterize. This point of view illuminates the richness of insight that understanding HRV can yield, and Grippo uses a correspondingly vast bibliography to support her exposition.

Readers may find the following organizational framework a helpful guide through the remaining variety of papers about the centrality of HRV in a systems perspective on health and well-being. The articles can usefully be grouped into three sections related inquiry: (1) What are we measuring when we measure HRV and how do we measure it? (2) What do HRV measurements tell us? (3) HRV interventions to promote behavioral health and treat behavioral disorders.

Section (1) includes the two articles by Ernst, the overview of HRV quantitative measures by Shaffer and Ginsberg, and the article by Davila et al. describing the validation of the PhysioCam by an exciting and potentially game-changing new method of heart beat detection and analysis. In the first of his two articles (“More than Heart Beats”), Ernst returns us to the origins of modern scientific appreciation of HRV with citations from classic work of the 1980's and 1990's (e.g., Axelrod, deBoer, Karemaker, and Armor) and even back to the 1970's, In the second of the two joined-at-the-hip articles (“Hidden Signals”), we go even farther back in time, to antiquity and traditional eastern medicine then jumping forward to nineteenth century medical studies of blood pressure, heart rate, and respiration before electronic apparatus existed. The bibliographies of Ernst's two articles along with the articles themselves might be thought of as an essential collection of readings for the scientist, researcher, and clinician interested in understanding HRV. The article by Shaffer and Ginsberg on the nature and collection of quantitative aspects of HRV has proven to be hugely popular, with over 8,600 views as of this writing, a quantitative indicator of the interest that studies of HRV are generating. The development of the PhysioCam, documented in the article by Davila et al., is intended to overcome the limitations of contact sensors for ambulatory recording of heart rate. The PhysioCam is capable of measuring arterial pulse from a distance with sufficient precision to derive HRV during different challenges, using an off-the-shelf passive digital color video camera sensor to extract arterial pulse from the slight variations of light reflected from an individual's face. Still in development, the PhysioCam is a technology that allows for unobtrusive monitoring of heart rate, which will have many applications for furthering understanding of the role that HRV and autonomics plays in everyday life and which we will surely be hearing much more about.

Section (2) is comprised of five articles placing HRV in the context of human physiology system function and dysfunction. The experience of threat-related autonomic responses, and their adverse effects on health, is placed in a bio-evolutionary framework in the paper by Kolacz and Porges. Their analysis is grounded in the neural architecture integrating cardiac autonomic control, pain, and the gastrointestinal tract, so in this way the functional gastrointestinal disorders (FGID's) can be seen as part of bio-psycho-social adaptation. Hage et al. considered the evidence for autonomic nervous system dysregulation in bipolar depressed patients, using HRV. Their question is an extension of the previously established observations that lower HRV accompanies unipolar depression. The experimental design called for random assignment to one of two medication groups (escitalopram + celecoxib or escitalopram + placebo), and included a healthy control group. They found evidence that patients had lower HRV power and higher heart rate pre-treatment, with not discernable effects of medication treatment on the cardiac measures. Their work is part of a larger movement to explore the potential diagnostic and prognostic value of HRV and other psychophysiological variables as biomarkers for psychotropic treatment outcome of mental disorder in adults. Borelli et al. contributed a study of “Reflective Functioning” in children. Reflective functioning is based on introspective processing of emotional experience. They used a protocol designed to evoke reactions regarding the experience and expression of attachment-related needs which they correlated with attachment coded from semi-structured interview. Their important, if not unsurprising, finding was that RF was associated with lower cardiovascular reactivity and better recovery, mediated by level of attachment (more attachment, better cardiovascular function). This work appears to be a significant contribution to developmental theory of physiological emotion regulation in children. Psychophysiological reactivity on a body-system scale in active duty military personnel was reported by Haufler et al. to be a significant predictor of performance-related adaptive behavior in a standardized, simulated operational environment. The implications of this type of thorough clinical research for fitness and readiness (and not limited to soldiers) is enormous. The commentary article on HRV and Dentistry by Drury and Simonetti is novel in showing that “gentle dentistry” as it is commonly called today, is based on an understanding of the cardiovascular and neuroendocrine elements of response to stressful dental treatments. The piece has, and has a surprisingly deep bibliography to support it and is of interest in pointing the direction for future development of HRV indicators of situational health status.

The five articles of section (3) focus on ways the autonomic cardiac control system can be managed to achieve desired health outcomes. The first article is a laboratory investigation into the inter-relations between HRV, blood pressure, and mood performed by Steffen et al.. The researchers use quantitative indicators in a carefully controlled experiment. The responsivity of this nexus of adaptive functioning to simple but controlled perturbation by slow (“resonant frequency”) breathing tells us a lot about the tools people can use to successfully manage themselves in their ecological niche. Kirby et al. write an article that delves into the modulation of social engagement by the ventral vagal nerve control of HRV. This work describes a procedure to train people to better experience compassion and thereby improve social engagement and its psychophysiological correlate. The same idea is pursued in greater anatomical depth in relation to the use a “a non-invasive, computer-guided, acoustic stimulation neurotechnology” called closed-loop allostasis, reported by Shaltout et al. This technique led to notable improvements in HRV and most impressively, baro-reflex sensitivity (BRS) and several measures of arerial blood pressure, a major effector of systemic blood pressure homeostasis that is closely related to heart rate. Significant decreases in self-reported insomnia and depression were also recorded. By measuring regional brain electrical activity, a trend for improved balance of temporal lobe high frequency amplitudes was noted. Non-invasive vagal nerve stimulation is a rising alternate treatment technique to produce increases in vagal tone and parasympathetic function to address the behavioral and emotional symptoms of sympathetic hyper-arousal. In their article, Lamb et al. publish their data in 22 combat Veterans. These clinical researchers collected autonomic physiological reactivity data at baseline and after transcutaneous vagal nerve stimulation (tVNS). Veterans were classified as either mTBI-PTSD or healthy and randomized to stimulation or sham control conditions. The tVNS intervention was well-tolerated and results suggested that effects did occur on systems that modulate emotional regulation. In closing, McCraty is a well-known person throughout the HRV community, having been a proponent of HRV Biofeedback for decades. His experience in the field can be traced to the very roots of awareness of the power and plain excitement of HRV engagement. Among his many areas of study and advocacy can be found the concept of “social coherence.” These ideas springboard off simple group HRV Biofeedback infused with the basic scientific notions of social nervous system and its role in social engagement a la Porges' polyvagal theory, past the newly emerging field of scientific study of interoception, and lands in the field of electromagnetic potentials in the evolutionary dynamics of ecosystems. Sound thinking prevails in the article's central thesis that feedback of individual and group HRV will increase group cohesion, thereby promoting pro-social behaviors, such as kindness and cooperation among individuals, improved communication, and decreases in social discord and adversarial interactions. “Biomagnetic fields produced by the heart may be a primary mechanism in mediating HRV synchronization among group members” he writes. Peripheral, implicit, and embedded in this message is the “Global Coherence Initiative” (GCI). GCI takes social coherence to its farthest limits and into the frequency zone that is shared by solar-geomagnetic field synchronization and Schuman Resonances, where it has been noted that these resonant frequencies directly overlap with those of the human brain and cardiovascular system.

## Author Contributions

All authors listed have made a substantial, direct and intellectual contribution to the work, and approved it for publication.

### Conflict of Interest

The authors declare that the research was conducted in the absence of any commercial or financial relationships that could be construed as a potential conflict of interest.

